# Gain-Switched Ho:YAP Laser with a 6.7 ns Pulse Duration at 2117 nm

**DOI:** 10.3390/s25030878

**Published:** 2025-01-31

**Authors:** David Goldfisher, Rotem Nahear, Neria Suliman, Yechiel Bach, Salman Noach

**Affiliations:** Department of Electro-Optics, Jerusalem College of Technology, Jerusalem 9372115, Israel; goldidavid1@gmail.com (D.G.);

**Keywords:** SWIR laser, pulse laser, Ho:YAP, Tm:YLF, gain switch, passive Q switch, LIDAR

## Abstract

This study demonstrates, for the first time, an innovative gain-switched Ho:YAP laser designed for laser distance measurement and remote sensing applications. The laser operates at a wavelength of 2117 nm, well positioned within the short-wavelength infrared (SWIR) atmospheric transmission window of between 2.1 and 2.5 µm. The laser delivers short pulse durations of 6.7 ns, and pulse energies of 0.645 mJ, allowing for an enhanced range and improved resolution. The Ho:YAP laser is pumped by a passively Q-switched Tm:YLF laser, resulting in a compact and energy-efficient system suitable for various sensing applications in precise distance measurement and environmental gas detection.

## 1. Introduction

Laser systems operating in the 2 µm spectral range have gained significant attention for precise distance measurement and remote sensing applications. This wavelength range is particularly advantageous due to its “eye-safe” classification and high atmospheric transmission within the 2–2.6 µm window, making it ideal for applications in populated areas and long-range detection [1]. The strategic positioning of lasers within this spectral region, specifically around 2.1–2.5 µm, offers enhanced performance in critical sensing technologies, addressing both industrial and scientific needs [2].

Short laser pulses in the nanosecond range are vital for achieving high-precision distance measurement and remote sensing capabilities. Meeting the demanding requirements for long-distance detection necessitates laser systems capable of generating high-energy, short-duration pulses. In LiDAR systems and laser rangefinders, pulse duration directly impacts spatial resolution, with shorter pulses enabling more accurate time-of-flight measurements [3]. High peak power, coupled with substantial pulse energy, improves signal detection, increases the signal-to-noise ratio, and extends the capability to detect targets over extended distances. These pulse characteristics are particularly valuable for applications requiring precise long-range distance measurement and environmental monitoring. Furthermore, shorter pulses are advantageous in minimizing overlapping returns from multiple objects in a densely populated measurement environment.

For portable, battery-powered devices, shorter pulses enhance energy efficiency without a significant reduction in laser efficiency, enabling reductions in size and weight. This attribute is especially important in the development of compact systems for field deployment, where both portability and durability are critical. Additionally, the compatibility of 2 µm lasers with emerging technologies like autonomous vehicles and airborne platforms expands their utility.

The unique spectral properties of the 2 µm range offer additional benefits beyond distance measurement. The strong and well-isolated absorption features of carbon dioxide (CO_2_) and CH₄, coupled with reduced water vapor (H_2_O) interference, make these lasers valuable for trace gas detection and combustion diagnostics [4,5]. Applications span diverse fields, including gas spectroscopy, medical surgery, material processing, and serving as pump sources for mid-IR lasers, such as Cr:ZnSe and Cr:ZnS [2]. These capabilities position 2 µm lasers as essential tools for addressing complex challenges in monitoring industrial processes and ensuring environmental compliance.

Recent studies highlight the versatility of 2 µm lasers across scientific, industrial, and environmental fields. Here, we highlight two recently published review papers regarding this topic. The first, by Yang et al. (2021), describes advancements in diode-pumped solid-state lasers using active and passive modulators within this spectral range [6]. The second, by Li et al. (2023), focuses on the development of 2 µm solid-state lasers for LiDAR applications over the past decade [7]. Both reviews emphasize that future progress in sensing and LiDAR technologies hinges on the creation of novel laser sources capable of delivering high pulse energy and short pulse durations.

**Pulse Generation Techniques**

Several methods exist for generating short laser pulses. The **Q-switch** method is the most commonly used, wherein the lasing process is temporarily inhibited, allowing the population inversion to surpass the lasing threshold. Once the inhibition is lifted, the stored energy is released as a short, intense pulse. The Q-switch method can be implemented by an active Q-switch mode applying an acousto-optic modulator (AO) or an electro-optic modulator (EO), or by a passive Q-switch mode using different saturable absorber materials (SA). This method can generate high-energy pulses, mainly through the active Q-switch methods; however, achieving pulse durations of just a few nanoseconds is uncommon [8]. Korenfeld et al. (2015) reported a 26 ns pulse duration and 4.22 mJ energy per pulse at 900 Hz PRF and a wavelength of 1908 nm using Tm:YLF as a gain medium and Cr:ZnSe as an SA [9]. Duan et al. (2019) reported a 20 ns pulse duration and 3.4 mJ energy per pulse at 5 kHz PRF and a wavelength of 2100 nm using Ho:SYSO as a gain medium [10]. Wang et al. (2019) reported a 7.5 ns pulse duration and 1.44 mJ energy per pulse at 1 kHz PRF and a wavelength of 2122 nm using Ho:YAG single-crystal fiber as a gain medium [11], both using an active Q-switch AO modulator. In the mentioned reference [2], the author reviewed twenty-seven publications on actively Q-switched pulsed lasers in the 2 μm range for LiDAR and sensing applications. Among these, only two references report pulse durations of less than 10 ns. Yao et al. (2014) demonstrated a short pulse duration of 4.7 ns with an energy of 0.9 mJ per pulse, utilizing an AO modulator and based on a Ho:GdVO_4_ crystal emitting at 2048 nm with a repetition rate of 5 kHz [12]. Qin et al. (2021) achieved a pulse duration of 4.6 ns and an energy of 0.9 mJ per pulse using an EO modulator with the same crystal, emitting at 2100 nm with a repetition rate of 5 kHz [13].

The implementation of this method necessitates the inclusion of an active modulator, such as an acousto-optic (AO) or electro-optic (EO) device. This addition impacts the system by increasing its complexity, size, and cost, as well as requiring a dedicated driver for the modulator. These factors must be carefully considered when designing laser systems intended for applications where compactness and cost-efficiency are critical.

Another approach, **cavity dumping**, involves rapidly reducing the reflectance of the output coupler to release the stored energy from the cavity. This method produces very short pulses but is limited by the shorter photon lifetime compared to the upper-state lifetime of the gain medium. This concept was first demonstrated in an Nd:YAG laser, showing that an energetic pulse was limited only by the cavity length of the laser [14], where pulse lengths are generally dictated by the round-trip time of a photon within the cavity (2 Lc/c, where Lc is the cavity length). To facilitate cavity dumping, a fast intracavity element must be used to rapidly dump the stored energy within the cavity. For example, an AOM can be used to deflect the output beam; alternatively, a Pockels cell inducing a rapid polarization change in combination with a polarization-selective output coupler is an effective fast dumping element. A dumped cavity setup is more complicated to align than simple Q switching and may require a control loop to choose the best time to dump the beam from the cavity.

A demonstration of the method in the 2 µm range, published by Zhang et al. (2016), achieved a 4 ns pulse duration in an EO (Pockels cell) cavity-dumped Ho:YAG (Holmium doped yttrium aluminium garnet) oscillator, with energy per pulse of 126 µJ and a peak power of 31.5 kW at a repetition rate of 100 kHz [15]. The same author also reported a 3 ns pulse duration and 12.3 µJ energy per pulse at 100 kHz PRF at an emitted wavelength of 2012.7 nm using a Tm:LuAG crystal as a gain medium [16]. Duan et al. (2018) reported a 3.6 ns pulse duration and 60 µJ energy per pulse at 100 kHz PRF and a wavelength of 2100.5 nm, using a Ho:SSO as a gain medium [17].

More recent work by Li et al. (2023) described a langasite (LGS) EO, Ho:YAG cavity-dumped laser. A constant pulse duration of 7.2 ns was achieved, with energy per pulse of 430 µJ at a repetition rate of 100 kHz [18]. Although few-nanosecond pulse durations can be achieved using this method, the energy per pulse values appear to be relatively low.

The **gain-switch method** provides a compelling alternative, particularly for materials with short upper-state lifetimes where Q switching becomes impractical. In this technique, the pump source is pulsed, creating high-energy, with short-duration laser pulses. Gain switching offers additional advantages, such as enabling the use of a shorter cavity, as it does not require the inclusion of Q-switch elements like AO modulators or SA. Shorter cavities are critical for minimizing pulse durations, as the duration is linearly proportional to the cavity round-trip time. However, gain switching for sensing applications in the 2 µm range is uncommon due to the complexity of generating suitable pulsed pump sources.

Among the few studies implementing the gain-switch method in the 2 µm spectral range, Tang et al. (2010) obtained a 20 ns pulse duration and 4.1 mJ energy per pulse at 2 kHz PRF and a wavelength of 2130 nm using a Ho:LuAG crystal as a gain medium [19]. Zhang et al. (2016) achieved a 44 ns pulse duration and 90 µJ energy per pulse at 20 kHz PRF and a wavelength of 2089 nm using a Ho:YAG crystal as a gain medium [20]. These results for gain switching are promising; however, they are very similar to the results obtained in the Q-switch method.

Breakthrough progress in this field was reported by Bach et al. (2022) from our laboratory. He demonstrated a Ho:YAG gain-switched laser with a pulse duration of 3.35 ns, 0.7 mJ pulse energy, and a peak power of 209 kW at a wavelength of 2090 nm, pumped by a passively Q-switched Tm:YLF laser [21]. These results highlight the potential of this method and the need to apply it to other crystals, such as Ho:YAP as described here, to expand the laser sources, in this spectral range, necessary for advanced sensing and LiDAR applications.

The Ho:YAP (holmium-doped yttrium aluminum perovskite) crystal is a promising material for tunable and short-pulse lasers in the 2 µm range, typically emitting at 2118 nm when pumped at 1910 nm or 1940 nm [22]. Ho:YAP exhibits several advantages over other commonly used laser crystals such as Ho:YAG. One key advantage is its natural birefringence, which results in linearly polarized output with virtually no depolarization loss [23].

Ho:YAP also has a broader absorption bandwidth, of nearly 1.9 µm, compared to Ho:YAG, with multiple absorption peaks depending on the crystal orientation (e.g., 1883 nm, 1916 nm, and 1947 nm for b-cut crystals). This characteristic provides great flexibility for in-band pumping using various Tm-doped laser sources, ensuring efficient energy transfer and minimizing quantum defect heating [22,24].

Compared to Ho:YAG, Ho:YAP has a larger stimulated emission cross-section (approximately 0.82 × 10^−20^ cm^2^) and a longer fluorescence lifetime (about 6 ms for the ^5^I_7_ → ^5^I_8_ transition). These properties contribute to achieving high slope efficiencies and significant pulse energies in Q-switched and mode-locked regimes [22].

Due to its high efficiency, natural polarization, and robust thermal properties, Ho:YAP is particularly well-suited for applications in lidar, atmospheric sensing, and medical devices requiring precise, high-quality laser beams [22,24].

Recent studies have demonstrated the potential of Ho:YAP for various applications. Table 1. summarizes the state-of-the-art results reported in this field during recent years. It can also be seen that most of the short-pulse generation efforts have relied exclusively on the Q-switch method. The table shows the pulse duration range and other laser characteristics achieved with different active Q-switch configurations. It can be seen that a maximum energy per pulse of 1.7 mJ is achieved with a pulse duration of tens of ns.

Building on these findings, our work demonstrates the first gain-switched Ho:YAP laser implementation. By optimizing cavity and pump parameters, we achieved few-nanosecond pulse durations, with reasonable values of energy per pulse suitable for advanced capabilities in sensing and Lidar applications. Our achieved results highlight the potential of gain switching as a scalable approach to meet the demands of future laser technologies.

## 2. Method

**Scheme 1:**
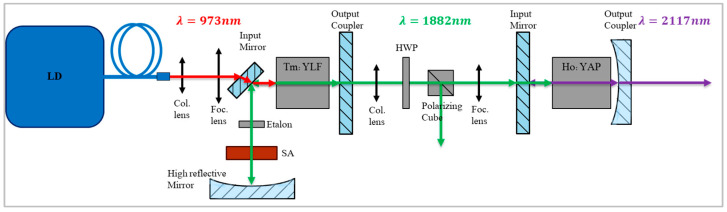
Experimental setup schematic of the external Ho:YAP laser and its Tm:YLF fundamental pump laser.

In this work, an implementation of a gain-switch method, using a pulsed Tm:YLF as a pump source and the Ho:YAP gain-switch external cavity, is presented. The laser design is depicted in Scheme 1. The laser system includes an L-shape, pulsed Tm:YLF laser as a pump source, and a linear Ho:YAP gain-switch external cavity. The pump source was based on 15 mm-long 3-at.% Tm:YLF crystal, end-pumped by 793 nm, 30 W laser diode, and passively Q-switched by Cr:ZnS. This design shortens the cavity length compared with using an active Q switch and, therefore, shortens the pulse duration and eliminates the need for another electric circuit as well. The Tm:YLF total cavity length was 142 mm. The L-shaped design the Tm:YLF utilizes to decrease the weight and volume of the whole gain-switch laser system, makes it suitable to a variety of portable platforms for 3D sensing or mapping. The Tm:YLF laser wavelength was tuned using a 100 µm thick etalon to 1882 nm, the Ho:YAP absorption peak. The goal of the tuning was to improve the Ho:YAP efficiency.

The Tm:YLF beam was collimated and focused on the Ho:YAP crystal to a spot diameter of 240 µm. The Tm:YLF linearly polarized beam [29] was used advantageously by aligning a polarizing cube with the Tm:YLF beam polarization, and by placing a half-wave plate (HWP) in front of the polarizing cube. In this way, by rotating the HWP and the beam polarization, the beam polarization is not parallel with the polarizing cube, and the orthogonal element of the beam is reflected out of the system. Thus, the HWP and the polarizing cube were used as an attenuation system. (The HWP and the PBS cube are not required in a final gain-switch system; they were used in the current setup for measurement and characterization purposes. Therefore, there is potential for a further reduction in the system dimensions beyond what is demonstrated here.)

The Ho:YAP cavity was composed of an input plano mirror, a 10 mm long 1.05% Ho:YAP b-cut crystal, and an output coupler with a radius of curvature of 50 mm with 60% reflectance for the laser and full reflectance for the pump, which increased the Ho:YAP absorption. The Ho:YAP total cavity length was 15 mm, and the resonant mode size was estimated by the ABCD method to be ∼178 µm diameter in the gain medium.

In all the following measurements, the output power measurements were done with a power meter (A-35, Ophir Optronics, Jerusalem, Israel). The pulse energy was measured with an energy meter (Ophir, PE50-C). Pulse temporal characterization was performed using an extended InGaAs fast photodetector with 28 ps rise-time (ET-5000, 12.5 GHz, Electro-Optics Technology (EOT), Traverse City, MI, USA) and a 1 GHz oscilloscope (Agilent, MSO7104A). The laser spectrum was acquired by an extended InGaAs 1D array spectrometer (BaySpec, BaySpec Inc., San Jose, CA, USA). Spatial profiling of the laser beam was performed using a pyroelectric camera (Pyrocam III-HR, Spiricon (Ophir Optronics), Jerusalem, Israel).

## 3. Results & Discussion

In this section we will present the results of the gain-switch laser and discuss their implications.

The Ho-Yap gain switch was pumped by a pulsed Tm:YLF laser emitting at 1882 nm, with maximum energy/pulse of 1.66 mJ at a PRF of 4.9 kHz, and with 31 ns pulse duration for the highest pumping power of 30 W, corresponding to a slope efficiency of 31.9% and an optical-to-optical conversion efficiency of 27.3%. The Tm-YLF wavelength was tuned to this fixed wavelength to align it with the Ho:YAP absorption peak, in order to improve the Ho:YAP efficiency.

**Figure 1:**
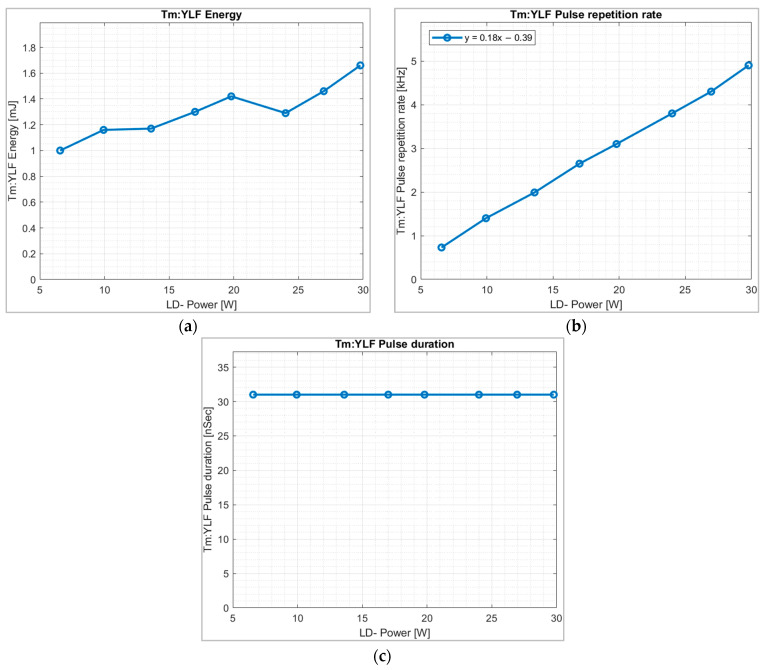
(**a**) Tm:YLF Energy versus LD-Power. (**b**) Tm:YLF Pulse repetition rate versus LD-Power. (**c**) Tm:YLF Pulse duration versus LD-Power.

The graphs show the typical behavior of a passively Q-switched laser. It can be seen that, with increasing pump energy, the pulse energy changes slightly from 1 to 1.6 mJ (Figure 1a), while the PRF changes significantly from 0.8 to 5 KHz (Figure 1b), and the pulse duration remains unchanged at 31 ns for all pumping ranges (Figure 1c). (All Tm:YLF performances were measured right in front of the Ho:YAP cavity.)

**Figure 2:**
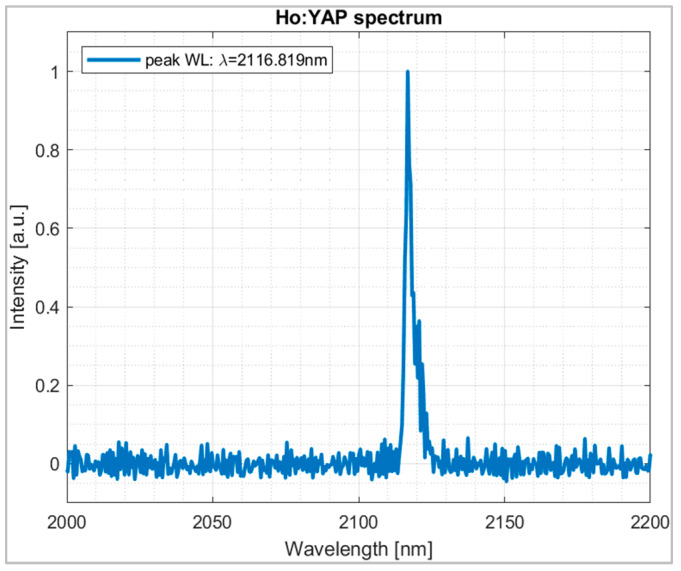
Ho:YAP laser spectrum recorded at the maximum output power.

The 2117 nm wavelength spectral emission of the Ho:YAP gain-switch laser is shown in Figure 2. The emitted wavelength was the same for CW and pulsed mode operation. (The spectral measurement for the CW mode was done by removing the SA).

**Figure 3:**
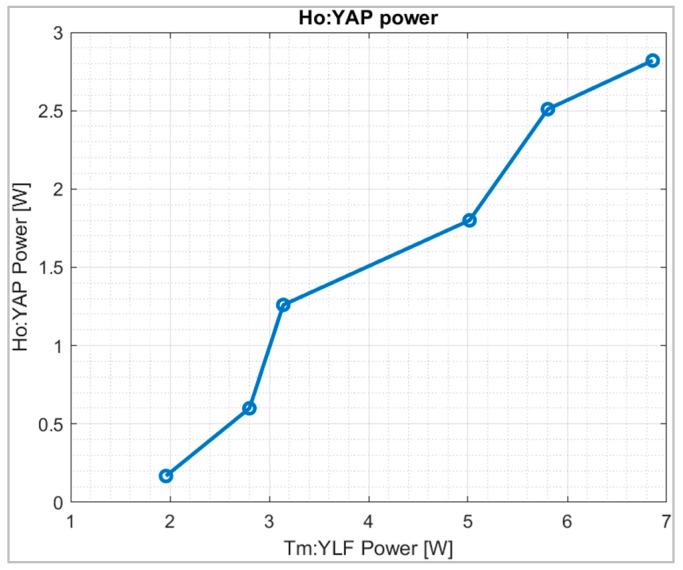
Ho:YAP output power versus incident pump power at CW operation.

Figure 3 demonstrates the Ho:YAP power versus the Tm:YLF pump power. At the threshold, the Ho:YAP laser emitted 2.82 W for the 6.86 W input from the Tm:YLF laser, corresponding to a slope efficiency of 54.1% and an optical-to-optical conversion efficiency of 41.1%.

**Figure 4:**
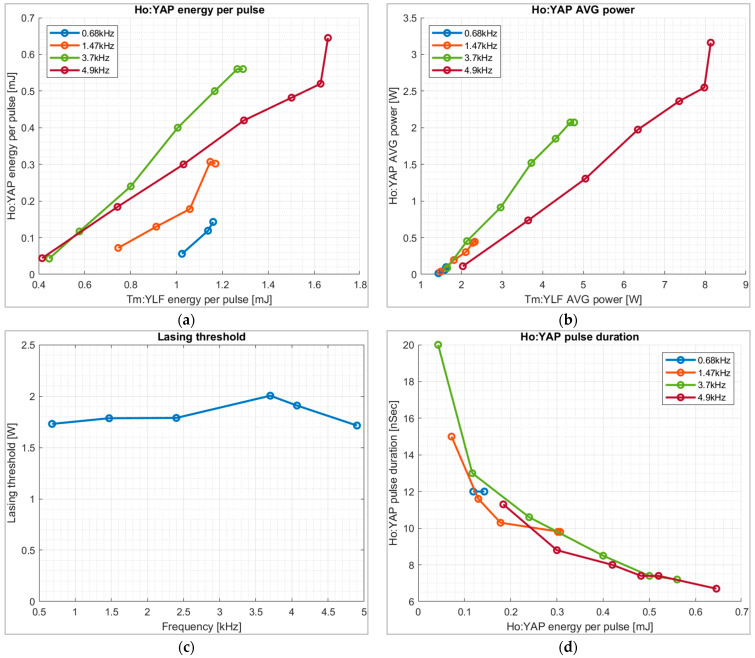
(**a**) Ho:YAP energy per pulse versus Tm:YLF energy per pulse at different repetition rates. (**b**) Ho:YAP AVG power versus Tm:YLF AVG power at different repetition rates. (**c**) Ho:YAP lasing threshold versus Ho:YAP frequency. (**d**) Ho:YAP pulse duration versus Tm:YLF energy per pulse at different repetition rates.

The pulsed performance at different repetition rates is presented in Figure 4a,b,d. In pulsed mode at a repetition rate of 4.9 kHz, a minimal pulse duration of 6.7 ns and maximal pulse energy of 0.645 mJ have been obtained for 1.66 mJ of the Tm:YLF, corresponding to a slope efficiency of 39.3%. (The slope up to the penultimate sampling point in the graph [where a jump in the slope changed], shows an optical-to-optical conversion of 38.9%, and a peak power of 95 kW).

The average power of the Ho:YAP as a function of the average power of the Tm:YLF is presented in Figure 4b. It can be observed that the lasing threshold remains consistent across the different frequencies, around 1.8 W, as shown in Figure 4c. The slope efficiency is approximately 50%, with the lowest value at a frequency of 4.9 kHz (41%, excluding the final jump point), and the highest at 3.7 kHz (63.6%). The highest power obtained is 3.16 W at a frequency of 4.9 kHz, corresponding to an optical-to-optical conversion efficiency of 38.9%.

The graph of pulse width as a function of energy exhibits a characteristic behavior: at lower energies, there is a noticeable improvement in pulse width as energy increases. However, as the energy continues to grow, the response diminishes, and the graph becomes asymptotic at higher energies. This trend is consistent with similar findings reported in the work of Shaul Avichai Golan et.al [30].

**Figure 5:**
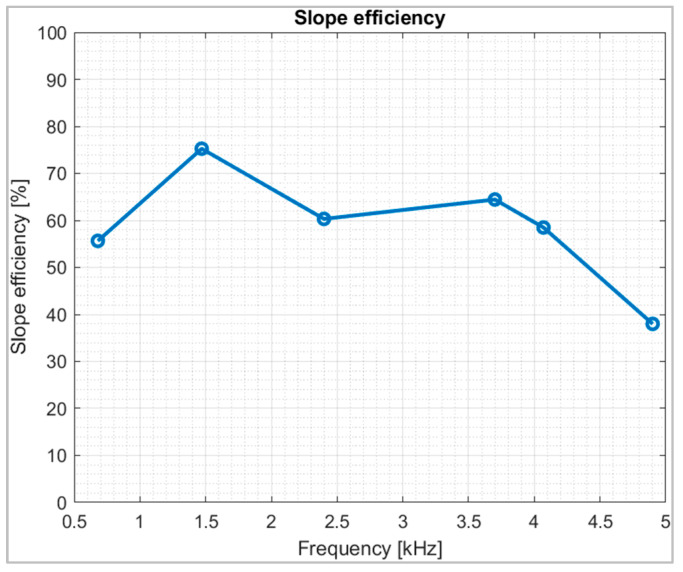
Ho:YAP Energy slope efficiency versus Ho:YAP frequency.

It can also be observed (Figure 4d) that, for all frequencies, the graphs overlap as energy increases. Additionally, the graph loses its slope as the energy increases, indicating that further increasing the energy at some point yields minimal improvement in pulse width. In Figure 4a, for a frequency of 4.9 kHz, it can be observed that the highest energy point deviates from the linear trendline of the graph’s slope. This phenomenon was consistently observed across various measurements and is likely due to thermal effects causing shifts in the resonance of the Ho:YAP laser. Examining Figure 4d, it is evident that the energy jump point also aligns well with the graph depicting pulse width as a function of energy. The slope efficiency ranged between 50% and 65% at most frequencies, peaking at 75% at 1.47 kHz. From a frequency of 3.7 kHz, there is a noticeable decrease in slope efficiency, reaching 38% at 4.9 kHz (Figure 5). This behavior can likely be attributed to the changes in the crystal’s response due to thermal effects.

The optical-to-optical conversion efficiency was 39% at 4.9 kHz, peaking at 43.5% at 3.7 kHz, and dropping to 12% at 0.68 kHz.

**Figure 6:**
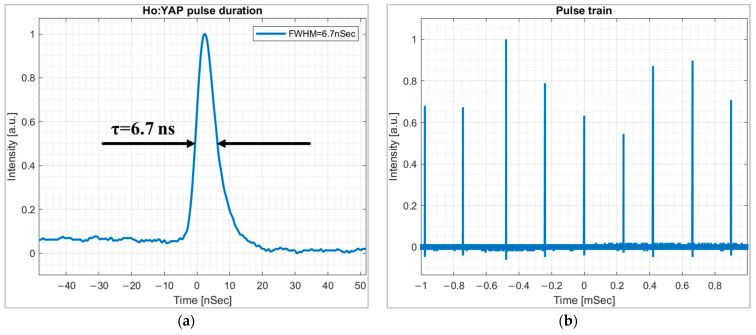
(**a**) Ho:YAP temporal profile of a 6.7 ns laser pulse. (**b**) Ho:YAP pulse train at 4.9 kHz.

The pulse temporal profile, presented in Figure 6a, where a short pulse of 6.7 ns FWHM was measured. The pulse train at 4.9 kHz is presented in Figure 6b. The maximum standard variation (STD) of the Ho:YAP pulses was measured to be 19% of the average pulse energy. The relatively high variations can be explained by variations in Tm:YLF which are reinforced in Ho:YAP since the slope efficiency is much bigger than the optical-to-optical conversion.

The apparent variation in Figure 6b is higher than the measured STD. The reason for the higher noise level in the graph is the under sampling of the scope (250 MHz, 4 ns interval) that does not fit for 7 ns pulses.

**Figure 7:**
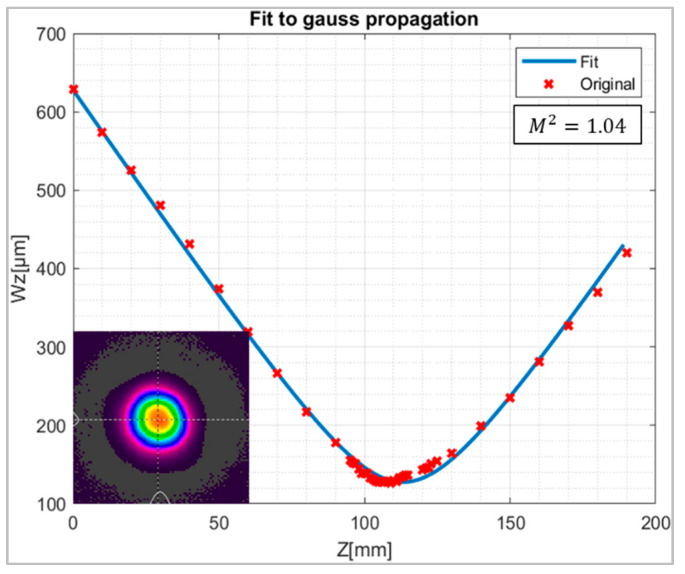
Ho:YAP beam quality.

The beam quality measurements of the Ho:YAP laser, presented in Figure 7, shows the beam radius variation along the *z*-axis and the fitted curve. The beam quality factors calculated as M^2^ = 1.04.

To summarize, in this study, we achieved promising results in terms of pulse duration and peak intensity of the lasing output at 2177 nm, contributing to enlarge the verity of the laser source of few ns with high energy at a new wavelength. However, we initially expected to surpass our former performance obtained with the Ho:YAG laser [22]. This expectation stemmed from the known relationship between the resonator length and the pulse duration. The rationale was that using a shorter Ho:YAP crystal would allow us to shorten the resonator length, thereby producing pulses shorter than 3.35 ns.

In practice, as can be deduced from Figure 4d, even with a significant increase in pulse energy, the asymptote converges to a limited pulse duration of approximately 6 ns. This result provides valuable insight of the gain-switched mechanism and challenges some conventional assumptions about pulse duration optimization.

To investigate this phenomenon, the direction of future research should be conducted as follows:

1. A systematic investigation using Ho:YAP crystals of varying lengths to explicitly map the relationship between crystal length and pulse duration. This study would help verify our hypothesis regarding the fundamental role of crystal length in determining minimum pulse duration.
2. An exploration of higher pump power regimes to better understand the asymptotic behavior of pulse duration. This investigation would help determine whether there exists potential for further optimization beyond the current 6 ns limit.

These proposed investigations could provide insights into the understanding of gain-switched lasers and, potentially, lead to strategies for achieving even shorter pulse durations in future designs. Understanding these limitations and their underlying mechanisms is essential for advancing the development of more efficient and higher-performing laser systems in this spectral range.

## 4. Conclusions

A highly efficient gain-switched Ho:YAP laser, with a few ns pulse duration and a high energy/pulse emission at 2177 nm, is demonstrated. The gain-switched laser, pumped by a Tm:YLF laser, demonstrated a 39% optical conversion efficiency, a 39.3% slope efficiency, and 3.16 W maximum average output power. Output pulse durations and energies/pulse were strongly dependent on the repetition rate. The shortest pulse (6.7 ns) and the highest energy (0.645 mJ), at 4.9 kHz, provided sufficient peak power (95 kW) for the Lidar sensing applications, and an efficient nonlinear frequency conversion to near-IR or mid-IR. To the best of our knowledge, this is the first demonstration of gain switching in Ho:YAP crystal. Compared to the current state-of-the-art Q-switched Ho:YAP laser’s preference, the achieved gain-switch results (especially in the pulse duration and peak power parameters) highlight the advantages of this method for sensing applications.

## Figures and Tables

**Table 1:** An overview of pulsed Ho:YAP laser results.

Q-Switch Method	Wavelength [nm]	Pulse Frequency [kHz]	Pulse Energy [mJ]	Pulse Width [nSec]	Peak Pulse Power [kW]	Slope Efficiency [%]	Year	Reference
AO	2118	5	1.58	22	**71.8**	45.9	2012	[25]
AO	2119	1	1.58	590	**2.7**	60	2012	[26]
AO	2118	10	1.07	29	**36.9**	62.1	2014	[27]
AO	2118	10	1.7	24	**70.8**	63.2	2014	[28]
EO	2118	4	1	33	**30.3**	50.6	2020	[22]
EO	2118	2	1.5	23	**65.2**	50.6	2020	[22]

## Data Availability

Data are contained within the article.
